# Deciphering the porcine intestinal microRNA transcriptome

**DOI:** 10.1186/1471-2164-11-275

**Published:** 2010-04-30

**Authors:** Soroush Sharbati, Marc R Friedländer, Jutta Sharbati, Lena Hoeke, Wei Chen, Andreas Keller, Peer F Stähler, Nikolaus Rajewsky, Ralf Einspanier

**Affiliations:** 1 Freie Universität Berlin, Institute of Veterinary Biochemistry, Oertzenweg 19b, 14163 Berlin, Germany; 2 Max Delbrück Centrum für Molekulare Medizin, Robert-Rössle-Strasse 10, 13125 Berlin, Germany; 3 Max Planck Institute for Molecular Genetics, Department of Human Molecular Genetics, Ihnestrasse 73, 14195 Berlin, Germany; 4 febit biomed gmbh, Im Neuenheimer Feld 519, 69120 Heidelberg, Germany

## Abstract

**Background:**

While more than 700 microRNAs (miRNAs) are known in human, a comparably low number has been identified in swine. Because of the close phylogenetic distance to humans, pigs serve as a suitable model for studying e.g. intestinal development or disease. Recent studies indicate that miRNAs are key regulators of intestinal development and their aberrant expression leads to intestinal malignancy.

**Results:**

Here, we present the identification of hundreds of apparently novel miRNAs in the porcine intestine. MiRNAs were first identified by means of deep sequencing followed by miRNA precursor prediction using the miRDeep algorithm as well as searching for conserved miRNAs. Second, the porcine miRNAome along the entire intestine (duodenum, proximal and distal jejunum, ileum, ascending and transverse colon) was unraveled using customized miRNA microarrays based on the identified sequences as well as known porcine and human ones. In total, the expression of 332 intestinal miRNAs was discovered, of which 201 represented assumed novel porcine miRNAs. The identified hairpin forming precursors were in part organized in genomic clusters, and most of the precursors were located on chromosomes 3 and 1, respectively. Hierarchical clustering of the expression data revealed subsets of miRNAs that are specific to distinct parts of the intestine pointing to their impact on cellular signaling networks.

**Conclusions:**

In this study, we have applied a straight forward approach to decipher the porcine intestinal miRNAome for the first time in mammals using a piglet model. The high number of identified novel miRNAs in the porcine intestine points out their crucial role in intestinal function as shown by pathway analysis. On the other hand, the reported miRNAs may share orthologs in other mammals such as human still to be discovered.

## Background

One of the basic requirements for the development of higher taxa in animal phylogeny was the acquisition of complex gene regulatory networks. Besides alternative splicing, RNA interference (RNAi) is recognized to be one of the key regulators of the post-transcriptional eukaryotic gene expression. Animal miRNAs (~ 22 nucleotides) belong to the class of endogenous small interfering non-coding RNAs. They direct the regulation of gene expression by binding partially complementary target sites in the 3' untranslated regions (3'UTR) of mRNAs [1]. It was noticed early that especially the 5' end of miRNAs is important for the binding of target mRNAs [2], in particular nucleotides 2-8, sometimes referred to as the 'seed' [3]. MiRNAs have been identified in numerous vertebrates including primates, rodents and fish but also in invertebrates e.g. worms and flies as well as in viruses [4]. A large number of miRNAs is highly conserved among related species indicating their role in crucial cellular processes such as stress adaption and hormone signaling [5]. During evolution more and more miRNA families were added to metazoan genomes and once incorporated into regulatory networks, few shifts of the mature sequence have occurred. These observations led to the hypothesis that morphological complexity is in part directed by the acquisition of new miRNA families during animal phylogeny [6,7]. While several hundreds of human miRNAs are known, limited data sets are available for other species. According to miRBase Release 14.0 [8], the number of noted human and murine miRNAs exceeds 700 and 500, respectively. Beside these species, the coverage of the miRNAome from other mammals e.g. pig remains scarce. To date 77 porcine miRNA sequences have been deposited in miRBase Release 14.0 and recently 96 novel and conserved mature miRNAs were identified in a porcine tissue pool while only 11 precursor molecules were identified [9]. Another recent study also based on searching for conserved miRNAs resulted in 162 identified precursors in swine skeletal muscle [10].

Besides their importance in livestock production, pigs are of particular biomedical interest because of their phylogenetic relation to humans. In this context and because human intestinal samples are limited and valuable tissue is only available after ileostomies or colonoscopies, rodents have been introduced as common models for human gastrointestinal studies. But limitations like differences in intestinal morphology, enteric microbiota, body mass, life span and different food intake raise concerns about the suitability of the rodent model. Obvious differences between mammals concerning the degree of intestinal maturation at birth should be taken into account. Rodents are born after a short gestation period with a premature intestinal tract. In contrast, pigs are born after a long gestation showing advanced intestinal development at birth. Therefore, pigs are closer to human intestinal development during fetal and neonatal periods [11]. In addition, the porcine anatomy and the physiology of digestion are very comparable to those of humans [12] favoring the piglet model as a suitable animal model to investigate human gastrointestinal diseases [13,14] and to understand signaling pathways related to mucosal development. For example, a piglet model of dextran sodium sulfate (DSS)-induced colitis was used to successfully evaluate mucosal immunity and therapeutic responses in patients with inflammatory bowel disease (IBD) [15]. Apart from Crohn's disease (CD), ulcerative colitis (UC) is one of the major idiopathic IBD. Interestingly, Wu and colleagues have recently shown that miRNAs are dysregulated in humans with UC. While three miRNAs showed significantly decreased expression, eight others were significantly increased in patients with UC [16]. However, to better investigate and understand regulatory networks of such exemplified diseases in the porcine model, a deeper coverage of the intestinal miRNAome is needed. Recently, we have reported 10 novel porcine miRNAs after introduction of a new concatameric cloning strategy [17]. Deep sequencing enables the immediate generation of a vast number of sequence data. As a proof of principle that deep sequencing can be used to discover hundreds of novel miRNAs from a single sample, we have introduced a method based on downstream sequence analysis using the algorithm miRDeep to detect ~ 200 previously unannotated canine miRNAs [18]. Thus, next generation sequencing methods provide the opportunity for detecting miRNA molecules at an unrivalled depth. In this study, we have applied deep sequencing followed by bioinformatical analysis of the acquired data to discover novel porcine miRNAs from a pool of porcine intestinal samples. After identification of novel porcine miRNAs including their precursors, the intestinal miRNAome was deciphered along the entire porcine intestinal tract including duodenum, proximal and distal jejunum, ileum, ascending and transverse colon.

## Results

### Deep sequencing unravels the expression of more than 300 potential porcine intestinal miRNAs

We have applied deep sequencing to analyze the expression of regulating miRNAs in the porcine intestine. For this purpose, a total RNA pool of samples from distal jejunum and ileum of four piglets at the age of 31 days was established in equal shares.

**Figure 1:**
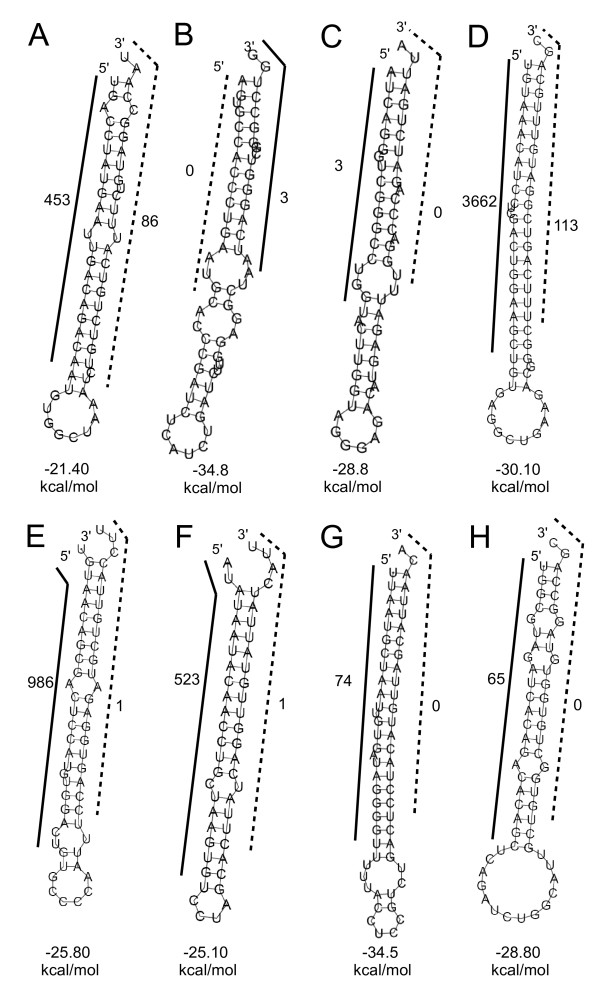
**Hairpin forming miRNA precursors**. The secondary structure of miRNA precursors is shown. The black bars indicate the mature miRNA, while the dashed bars indicate the star sequence. The numbers represent the deep sequencing reads of mature and star sequences, respectively. The minimal free energy (mfe) values are shown below. A) ssc-miR-215, B) MDM238, C) MDM392, D) ssc-miR-30a, E) ssc-miR-194, F) ssc-miR-374b, G) ssc-miR-155, H) ssc-miR-4331.

In total 5,745,733 reads were generated by deep sequencing of the small RNA fraction (20-30 nt). Of those 1,422,397 reads mapped to the pig genome perfectly. After specific selection (see material and methods section) of eligible reads, miRNA predictions were made using miRDeep, an algorithm to identify known and novel miRNAs in deep sequencing data. miRDeep uses the mapped reads as guidelines for excising potential miRNA hairpins from the genome. Each hairpin is assigned a score, which reflects the probability that the hairpin is a genuine miRNA precursor [18]. Altogether, 399 were scored by miRDeep and chosen for further analysis, of which 103 were known miRNAs (according to miRBase 14.0 and Nielsen et al. 2009). 354 hairpins were reported by miRDeep passing the cut-off score of -3, of which 140 were estimated to be false positives, representing a signal to noise ratio of 2.5. Hairpin forming precursors (including the flanking region) predicted by miRDeep exhibited low minimal free energies (mfe) ranging between -67.4 and -21.5 kcal/mol (Additional file 1). The hairpin structures of eight precursor sequences are exemplified in Figure 1 (for detailed information about all predicted hairpin forming precursors see Additional file 1). Among them were novel porcine miRNAs (Figure 1, H) that possessed low or no conservation among other mammals but also four putative viral miRNAs. Because of the imperfect coverage of the pig genome assembly an additional search of conserved mammalian miRNAs was performed. This approach yielded 119 porcine mature miRNA candidates that did not map to the pig genome assembly and 18 mature miRNA candidates that did map to the genome but had not been reported by miRDeep. Blast searches against known miRNAs (miRBase 14.0 and Nielsen et al. 2009) revealed 20 novel and conserved mature miRNAs (for details see Additional file 2).

### Microarray experiments verify the expression of 194 novel miRNAs

We have used the newly identified potential miRNA sequences as well as known porcine and human miRNA sequences to perform the first miRNAome analysis along the entire intestinal tract of a mammalian organism by using customized microarrays (febit biomed GmbH, Heidelberg, Germany). This approach served also as verification of miRDeep predicted data. Pooled samples from different intestinal loci (duodenum, proximal and distal jejunum, ileum, ascending and transverse colon) of four healthy 31 days old piglets were subjected to miRNA microarray analysis. MIAME-compliant data relative to the applied platforms as well as processed and raw sample data were submitted to the NCBI GEO repository [19] and accession-numbers were assigned (platform and samples for miRDeep predicted probes: platform, GPL9724; duodenum, GSM501150; proximal jejunum, GSM501151; distal jejunum, GSM501152; ileum, GSM501153; ascending colon, GSM501154; transverse colon, GSM501155; common reference, GSM501156; platform and samples for human samples including porcine conserved mature miRNA: platform, GPL9724; duodenum, GSM501118; proximal jejunum, GSM501119; distal jejunum, GSM501120; ileum, GSM501121; ascending colon, GSM501122; transverse colon, GSM501123; common reference, GSM501124). For selection of highly reliable expression data and validation of the miRDeep prediction, we decided to apply a stringent selection strategy. The predicted mature as well as relating star sequences were synthesized in seven replicates on a febit biochip. After background subtraction and variance stabilizing normalization (VSN) of array data, only expression values of mature sequences higher than 7 were considered. This threshold is chosen as a cut-off for selecting bona fide signals. From the eliminated data also those were considered with a signal intensity of the star sequence higher than 7. In our microarray experiments, we detected 31 miRNAs showing consistently higher signal intensities of their star strands compared with the predicted mature molecules (Additional file 3). After choosing this high level of quality assurance by simultaneous detection of the mature as well as star sequence we have verified the expression of 194 miRDeep predicted mature miRNAs. We considered another 7 sequences as genuine miRNAs, which showed no signal on microarrays but were conserved among animals. Consequently, we have identified 201 apparently novel porcine miRNAs out of 399 miRDeep predicted sequences (Additional file 3). As mentioned above 354 predicted precursors possessed a score above the cut-off -3, of which an estimated 140 were false positives. According to the microarray experiments, we have identified 95 false positive miRNAs representing a signal to noise ratio of 4.2 and underlining the robustness of the miRDeep prediction.

**Figure 2:**
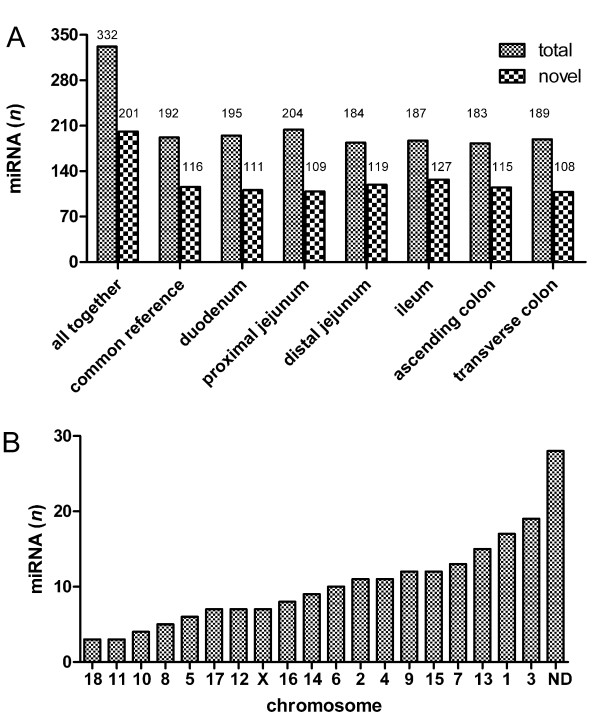
**Number of identified miRNAs**. A) The total number (small squares) and the number of apparently novel porcine miRNAs (large squares) are given for all intestinal loci including the common reference. B) The number of the assumed novel miRNAs possessing predicted hairpin forming precursors per chromosome. Abbreviation: ND, not determined.

By regarding also the identified conserved mature miRNAs not covered by miRDeep and the known porcine sequences deposited in miRBase, we have verified the expression of 332 miRNAs expressed in the porcine intestine including 201 apparently novel porcine miRNAs (194 miRDeep predicted plus seven conserved mature miRNAs) corresponding to 61% (Figure 2A). Taken together, between 204 (proximal jejunum) and 183 (ascending colon) miRNAs were found to be expressed in different intestinal loci, while the percentage of novel porcine miRNA varied between 53 and 68% depending on the locus analyzed (Figure 2A and Additional file 4).

**Figure 3:**
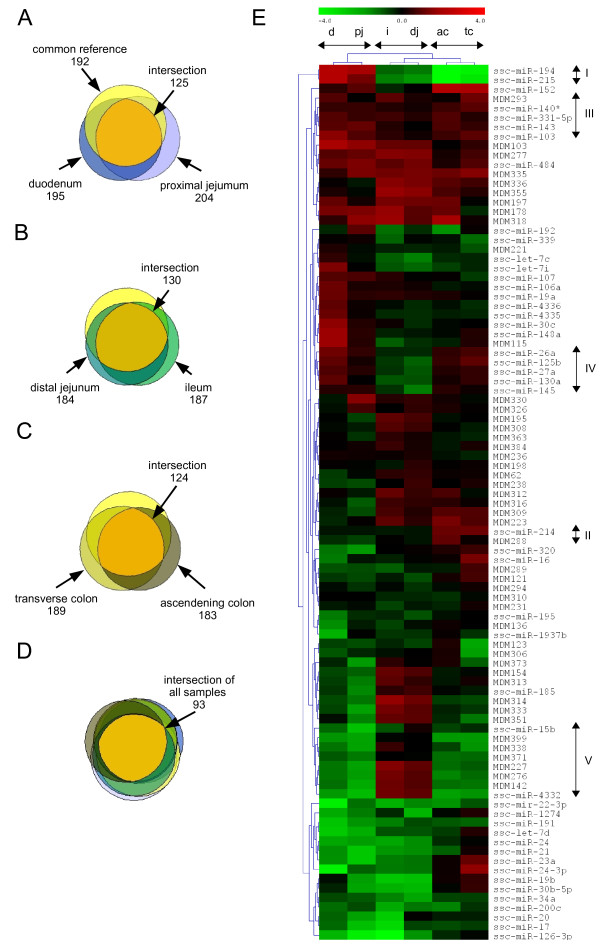
**Venn diagrams and hierarchical clustering of microarray expression data**. Venn diagrams (A-D) show the overlapping expression of miRNAs in related intestinal loci including the common reference. A) 125 miRNAs showed overlapping expression in duodenum, proximal jejunum and the common reference. B) The Venn diagram shows 130 miRNAs that were common in ileum and distal jejunum. C) 124 miRNAs showing overlapping expression were detected in both colonic loci. D) 93 miRNAs were commonly expressed in all analyzed samples. These miRNAs were chosen to perform the hierarchical clustering. E) Figure shows the heatmap of expression data after hierarchical clustering of both samples and genes. The heatmap is based on calculated log ratios between samples and the common reference. Arrows indicate clusters of interest both on gene as well as sample level. Abbreviations: MDMx, apparently novel miRNAs, d, duodenum; pj, proximal jejunum; dj, distal jejunum; i, ileum; ac, ascending colon; tc, transverse colon.

A high number of analyzed miRNAs showed overlapping expression in morphologically related intestinal loci shown by the Venn diagrams in Figure 3A-C. There was an overlapping expression of 130 miRNAs in distal jejunum and ileum (including the common reference) corresponding to 71% and 70% of totally expressed miRNAs in distal jejunum and ileum, respectively (Figure 3B). Consistent expression was also determined in both colonic loci, 124 miRNAs were expressed both in transverse and ascending colon representing 65% and 67% of regarding totally expressed miRNA (Figure 3C). For comparative expression analysis among the intestinal loci, we have applied the strategy of using a common reference representing a pool of all samples. As shown in Figure 3D, 93 miRNAs showed ubiquitous expression in all loci including the common reference. For evaluation of comprehensive microarray data and to allow for comparison of spatial miRNA expression, we have calculated the log ratios of those 93 miRNAs for every locus and their counterparts from the common reference. In consistence with the Venn diagrams, hierarchical clustering of both genes and samples revealed similar expression profiles in morphologically related intestinal tissues. Both colonic loci clustered together, they formed a superior group with the distal jejunum and ileum while duodenum and proximal jejunum clustered together (figure 3E). At the gene level we have identified several clusters, while five of which were of particular interest. Cluster I (Figure 3E) shown in the hierarchically clustered heatmap harbored only miR-194 and miR-215, which were highly expressed in the proximal parts of the small intestine (duodenum and proximal jejunum). Consequently, we searched for predicted targets using Targetscan [20] and subjected the outcome to pathway analysis using the tool DAVID [21] and considering the affected BioCarta and KEGG pathways. This revealed that these miRNAs seem to have an impact mainly on pathways that determine cellular differentiation and proliferation such as TGF-beta, ErbB, insulin and mTOR signaling pathways. Cluster II consisted of miR-214 and MDM288 showing increased levels of expression in both colonic loci. The downstream analysis indicated that cell cycle determining pathways such as Cyclin E and E2F destruction pathways but also cell to cell adhesion signaling were affected by these miRNAs. Cluster III harbored miRNAs that show ubiquitously high expression along the intestine. Two other clusters of miRNAs showed a decreased or increased expression in distal jejunum and ileum, respectively (Figure 3E, IV & V). The first one harbored known miRNAs such as miR-26a, miR-27a, miR-125b, miR-130a and miR-145 but the latter was mainly composed of novel and not conserved miRNAs. The miRNAs outlined in cluster V have predominant impact on intracellular signaling pathways such as MAPK/ERK, Notch and Wnt signaling but also on Mets affected macrophage differentiation and p53 signaling pathway.

In addition four miRNAs were identified, which partly matched known viral miRNAs. MDM133, 154, 329 and 335 may represent novel porcine specific viral miRNAs being partly identical to hsv1-miR-H1, rrv-miR-rR1-4, mdv2-miR-M18-5p and mdv2-miR-M30, respectively (data not shown). While MDM154 and MDM335 were expressed along the entire intestine, MDM329 was only detected in proximal jejunum and the expression of MDM133 was below the detection limit (Figure 3E).

**Figure 4:**
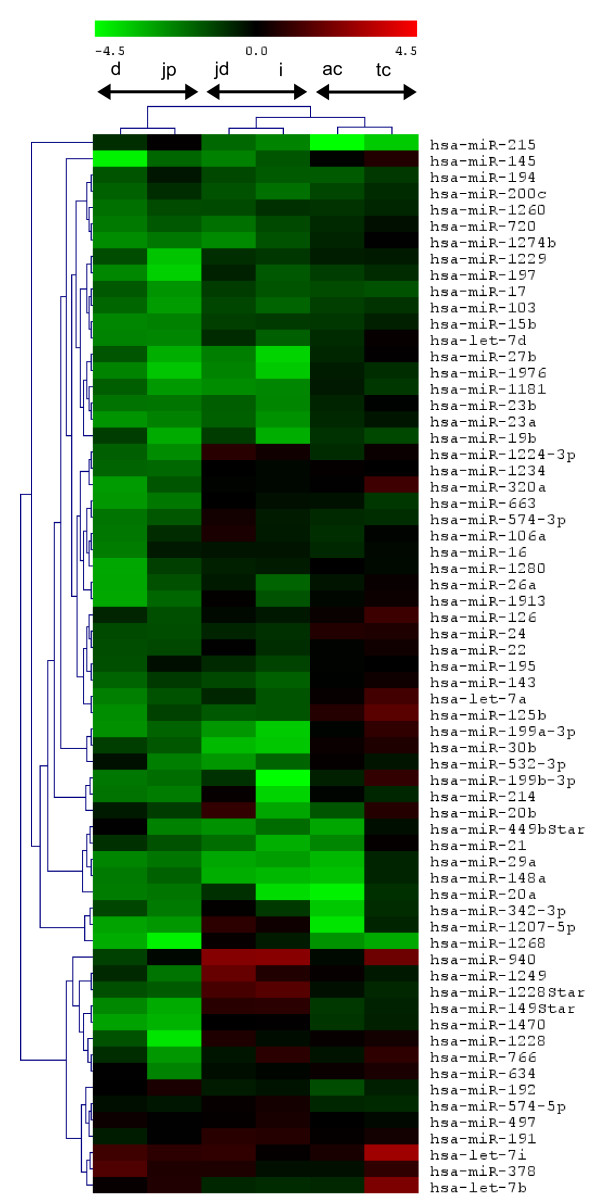
**Hierarchical clustering of the heterologous approach**. Heatmap of expression data is shown after hybridizing porcine samples versus human probes and hierarchical clustering of both samples and genes. Abbreviations: d, duodenum; pj, proximal jejunum; dj, distal jejunum; i, ileum; ac, ascending colon; tc, transverse colon.

In our microarray approach we also included known human miRNAs obtained from miRBase 13.0 allowing also a heterologous approach between human probes and porcine miRNAs. After selecting the miRNAs being expressed in all loci including the common reference, we have identified 66 miRNAs, which were chosen for clustering analysis as described above. Interestingly, there was consistent clustering of intestinal samples, as described for the homologous approach above. All related intestinal parts clustered together, while in concordance to the homologous approach the colon and the distal parts of the small intestine formed a superior cluster (Figure 4). Conserved miRNAs showed comparable expression patterns and clustering revealed consistency between both approaches, e.g. miR-126, miR-24 and miR-22 were combined in the same cluster and together with miR-16, miR-21 and let-7d showed increased expression in colon compared with other loci (Figure 4). After selecting miRNAs with consistent signal detection i.e. with overlapping detection in the homologous and heterologous approach (discarding of porcine specific sequences), 13 miRNAs were selected for correlation analysis between both approaches. The Pearson correlation coefficients were calculated depending on the locus and were between 0.7235 in duodenum and 0.9332 in ascending colon (p < 0.0001). The global Pearson correlation coefficient calculated from all loci was 0.7468 (p < 0.0001). The calculated local as well as general coefficients pointed to high linearity between both approaches (Table 1).

**Table 1:** Correlation analysis of microarray results.

	heterologous hybridizations vs. homologous hybridizations							
	global	duodenum	jejunum proximal	jejunum distal	ileum	**colon asc**.	**colon trans**.	common reference
**Pearson r**	0.7468	0.7235	0.9001	0.8731	0.8925	0.9332	0.8664	0.8155
	
**P value (two-tailed)**	< 0.0001	0.0052	< 0.0001	< 0.0001	< 0.0001	< 0.0001	0.0001	0.0007

**R^2^**	0.5577	0.5234	0.8102	0.7624	0.7965	0.8708	0.7506	0.6650

Correlation coefficients were calculated between heterologous hybridizations (human miRNA probes vs. porcine samples) and homologous hybridizations (porcine miRNA probes vs. porcine samples).

### Identified porcine miRNAs are chromosomally duplicated and partly clustered

After comparative expression analysis among the intestinal loci, we were interested if and how the identified miRNA precursors are organized within the porcine genome.

Therefore, we firstly sorted out only miRDeep predicted novel precursors whose expression was validated by microarray experiments in any of the intestinal parts or which were conserved among animals, respectively. Thereby, we have identified 201 apparently novel porcine miRNAs (Additional file 3). To find out the genomic location and organization of the identified precursors, sequences were blasted against the porcine high throughput genomic sequence (HTGS) database at NCBI http://www.ncbi.nlm.nih.gov/genome/seq/BlastGen/BlastGen.cgi?taxid=9823. As shown in Figure 2B, the chromosomal location of 86% of the identified miRNAs was determined. Most of the precursors were located on the chromosomes 3 followed by chromosome 1 (9 and 8%) and only 1% was organized in the chromosome 18 and 11, respectively. The chromosomal location of 14% of the precursors was not determined. Detailed information on genomic organization of the identified miRNAs is given in the Additional file 3. In addition to the identified genomic duplications and clustering of miRNAs, we were able to predict alternative precursors for the same mature miRNA (Table 2). Interestingly, miR-194 and miR-215 were both duplicated and located on chromosomes X and 10 and clustered within a 353 bp chromosomal region. Alignment of duplicated chromosomal regions revealed 100% identity of the nucleotide sequence (data not shown). The secondary structure analysis of this chromosomal site including both precursors revealed another hairpin structure, which was not covered by miRDeep and possessed the conserved seed of miR-488 (data not shown). The precursor of miR-421, for example, was located on chromosome X and was clustered with the known miRNA ssc-miR-374a within a distance of 107 bp, while no obvious hairpin was detected in between (Table 2). As shown in Table 2, joining hairpin forming precursors were predicted for several miRNAs. For example, two alternative hairpin forming precursors were predicted for the miRNA with internal IDs MDM238 and MDM392 (an ortholog of mmu-miR-2145). Both possessed low mfe (-34.8 and -28.8 kcal/mol) and were located within a fragment of 101 bp on chromosome 2 and may represent two alternative dicer substrates (Table 2 and Figure 1B and 1C).

**Table 2:** Porcine miRNAs with hairpin forming precursors and genomic locations.

conserved miRBase 14.0	internal ID	mature sequence	precursor sequence	mfe (kcal/mol)	gen. location	subject ID	start	end
*Genomic duplications*								
ssc-miR-194	MDM9	UGUAACAGCGACUCCAUGUGGAC	UGUAACAGCGACUCCAUGUGGACUGUGCCCCAAUUUCCAGUGGAGAUGCUGUUACCUU	-37.4	X	emb|FP565626.1|	43462	43519
					10	emb|CU466937.2|	6280	6223

ssc-miR-365-1	MDM48	UAAUGCCCCUAAAAAUCCUUAU	GAGGGACUUUUGGGGGCAGAUGCGUUUCCAUUCCACUAUCAUAAUGCCCCUAAAAAUCCUUAU	-33.8	3	emb|CU896561.2|	167752	167814
ssc-miR-365-2	MDM45	UAAUGCCCCUAAAAAUCCUUAU	GAGGGACUUUCAGGGGCAGCUGUGUUUUCUGACUCAGUCAUAAUGCCCCUAAAAAUCCUUAU	-35	12	emb|FP016089.2|	194688	194749

	MDM123	AAACGAUGAUGGAAGGUGCUGAGGA	CUCUCUGGUCCCUUCUCUCCCCUCUUCAUGGUUGACAGCAACCCCAGAAACGAUGAUGGAAGGUGCUGAGGA	-35.4	2	emb|CU896534.2|	31001	30933
	MDM266	AAACGAUGAUGGAAGGUGCUGAGGA	AAACGAUGAUGGAAGGUGCUGAGGAAGGGGUGCUAAUGUCCUAGCGUUUCCUCCUUCCAUUGUUAGGAGGC	-32.7	ND	ND	ND	ND

	MDM208	UCUGGCUGUGGUGUAGGCCGU	GGAUCUGCGCAUUGCCGCGAGCUGCAGUGUAGGUCACAGAUGUGGCUCAGAUCCAGCAUUGCUCUGGCUGUGGUGUAGGCCGU	-40.3	16	emb|CU571054.2|	59166	59247
	MDM116	UCUGGCUGUGGUGUAGGCCGUC	AGGUCACAAACACGGCUCAGAUCUGAUGUUGCUCUGGCUGUGGUGUAGGCCGUC	-41	13	emb|CU582976.2|	156369	156318

mmu-miR-1896	MDM214	AUGGGUGAGGAGUGUUGUGUAUAUA	AUGGGUGAGGAGUGUUGUGUAUAUAUAUUUGUGUGUUUGUAUAUACACAUCUCAAAGCUUAUUU	-27.2	6	emb|FP236156.2|	77046	76983

					16	emb|FP085480.3|	108843	108906

hsa-miR-1977	MDM262	GACUUGGAAUUAGUAGGGUGCUUA	GACUUGGAAUUAGUAGGGUGCUUAGAACAGUGCCUGAUACAUAGUUUCACAA	-26.2	4	emb|FP236645.1|	179755	179704
					14	emb|CT737346.2|	42310	42361

	MDM284	GCCGCGGCGUCCGGGCUG	GCCGCGGCGUCCGGGCUGUGUCCGCCCUGGCGGGCGCGCGGGGGCGGGAAGGCGCGGGCUGUCAAGGGCGCUGCGGCAG	-61	X	emb|CU466457.2|	83369	83334
					13	emb|CU582896.2|	20953	20988

*Genomic clusterings*								
ssc-miR-215	MDM1	UGACCUAUGAAUUGACAGACA	UGACCUAUGAAUUGACAGACAAUGUGGCUAAAUCUGUCUGUCAUUUCUGUAGGCCAAU	-29.8	X & 10	emb|FP565626.1|	43758	43815
ssc-miR-194	MDM9	UGUAACAGCGACUCCAUGUGGAC	UGUAACAGCGACUCCAUGUGGACUGUGCCCCAAUUUCCAGUGGAGAUGCUGUUACCUU	-37.4	X & 10	emb|FP565626.1|	43462	43519

ssc-miR-421	MDM86	AUCAACAGACAUUAAUUGGGCGC	CCUCAUUAAAUGUUUGUUGAAUGAAAAAAUGAAUCAUCAACAGACAUUAAUUGGGCGC	-35.8				
ssc-miR-374a	MDM23	UUAUAAUACAACCUGAUAAGUG	UUAUAAUACAACCUGAUAAGUGUUACAGCACUUAUCAGGUUGUAUUGUAAUU	-41.2				

bta-miR-2316	MDM279	CGGCCGGAGCCUGGACUGCUGC	GGCGCUAGGGAGCCAGGCCGGGGAGGACAGCUGCAGCCAGCCUGUCCUCCCGUCCGGCCGGAGCCUGGACUGCUGC	-63.5	15	emb|CU914511.2|	63040	62965
	MDM371	CGCGGGUGGGAAUGGGAAGG	CCCCCACCCCCGCCCCGGCUGACGCAAGAACCUGGCGGGCCGCGGGUGGGAAUGGGAAGG	-49.5	15	emb|CU914511.2|	63388	63431

*Alternatively predicted precursors*								
	MDM62	UGUGUCUGUGGCCUAGGCUGGCAG	UGUGUCUGUGGCCUAGGCUGGCAGGCAGCUGCAGCUCCAGUUCAACCCCUAGCCUGGGAACUUCCAUAUGCUCAGG	-36.4	9	emb|CU915614.1|	87273	87348
	MDM109	UGUGUCUGUGGCCUAGGCUGGCAG	GAGACAUGGUGUAGGUUGCAGACGUGGCUCAGAUCUGGCAUUGCUGUGUCUGUGGCCUAGGCUGGCAG	-46.9	9	emb|CU915614.1|	87229	87296

	MDM128	GCCGGAGCUGGAGGUGGG	GCCGGAGCUGGAGGUGGGCGAGGCCGCGGCACCCCCUCCGCUGCCGCCGCCGAGGGCCGCCUCCCUGGCCUCGGUGC	-52.7	17	emb|FP325255.2|	141939	141863
	MDM129	GCCGGAGCUGGAGGUGGG	CCUCUUCAGCGAGCGCGGCCGCUCCGUGCCCCUCGAGGAGCUGCCGGAGCUGGAGGUGGG	-50.4	17	emb|FP325255.2|	141981	141922

	MDM196	AGAGGUUCGGCUGUGCGGGCA	CUCCGCGGCGCUCGGAUAUCUGGCAGACCCGGGCGCGGGGAGGCUAGAGGUUCGGCUGUGCGGGCA	-50.6	3	emb|FP475960.2|	151377	151312
	MDM382	AGAGGUUCGGCUGUGCGGGCA	AGAGGUUCGGCUGUGCGGGCACUGCGCUCCGGGGACGGCCGCUGCACCUGC	-29.2	3	emb|FP475960.2|	151332	151282

mmu-miR-2145	MDM238	AUCAGGGUCGGGCCUGG	AGUGCCACCCUGAAUGCACCCGAUCUCAUCUGAUCUUGGAGGCUAAUCAGGGUCGGGCCUGG	-34.8	2	emb|CU929491.4|	132342	132283
mmu-miR-2145	MDM392	AUCAGGGUCGGGCCUGG	AUCAGGGUCGGGCCUGGUACUUGGUAGGGAGACAUGAGAUUUGGACCCAGAUCUGAUUA	-28.8	2	emb|CU929491.4|	132299	132241

The chromosomal organization of selected miRNAs that were identified by miRDeep either showing valid expression in microarray experiments or being conserved was determined by blasting against the porcine HTGS database at NCBI. Identified genomic duplications, clustering and alternatively predicted precursors are shown. Abbreviation: ND, not determined

## Discussion

Pigs represent an important model for biomedical research to study human disease, development or nutrition. As introduced, the porcine gastrointestinal model provides several benefits over other animal models such as rodents. Like humans, pigs are omnivorous implying very similar principles of the physiology of digestion and associated metabolic processes [12,22]. In mammals, the principal internal interaction between host and microorganisms or other antigens takes place in the intestinal tract. Because of the morphological as well as physiological similarities particularly the piglet intestinal model is suitable to study gastrointestinal disease both in adults and infants [13,14,23]. It is known that cellular differentiation is governed by miRNAs, for example it was recently shown that miR-145 directs the intestinal development in *Danio rerio *[24]. Moreover, Monzo and colleagues found a correlation between the miRNA expression profiles in human embryonic colonic mucosa and colorectal carcinoma. They found that the miR-17-92 cluster regulates cell proliferation pointing to the important role both in embryonic intestinal development and aberration of this epithelium [25].

In our study a model of weanling piglets at the age of 31 days has been used. As we have discussed in an earlier study, there are two waves of antigen exposure in a newborn mammalian organism: directly after birth and at weaning. The second is rated as an important period in the intestinal development that is characterized by massive exposure to food and microbial antigens followed by architectural reorganization of the small intestine [26]. It was reviewed that beneath the changes along the horizontal intestinal axis pronounced changes occur in the vertical axis (from crypts to villi) during intestinal development. This represents a gradient replacement of the cells of an immature phenotype with other cells that differ e.g. in transport functions. These changes are related to the rate of cellular turnover because there is a slow cell replacement at birth while an increase takes place at weaning together with a decrease of enterocyte lifespan [11]. Since miRNAs are key regulators of cellular proliferation and differentiation, we hypothesize that the weaning phase is accompanied by massive regulation of specific miRNAs governing the reorganization of the intestinal cellular response. Moreover, the randomization of our approach by using RNA pools of four piglets minimizes the bias in the miRNAome by possible biological variation.

In an earlier study, we have identified several porcine miRNAs applying a novel cloning strategy based on synthesis of concatamers [17] followed by expression profiling using a miRNA specific quantitative PCR approach [27]. Here a more straight forward approach was chosen based on new technologies to generate comprehensive data in a less time consuming manner. The validation of sequence data by means of customized microarray expression profiling allowed us not only to verify the generated porcine data, but also to include known human miRNA sequences. Since miRNA sequence data are not available in depth for all mammals, heterologous hybridization approaches may be the only alternative to perform expression studies in less common species. Supporting results from an earlier study [28] and the remarkable correlations between the heterologous (human probes versus porcine samples) and the homologous approach presented here, show that in phylogenetically close species the heterologous approach may be considered as a suitable alternative. However, the high number of novel and potentially porcine specific miRNAs discovered in this study argues against a heterologous approach.

We have identified several genomic clusters one of those including the porcine miRNAs miR-194 and miR-215. Braun and colleagues [29] have reported that these miRNAs also belong to a human cluster, which is induced by the tumor suppressor p53. It was shown that miR-192, miR-194 and miR-215 are highly expressed in normal colon tissue while their expression is dramatically decreased in colon cancer. They conclude that these miRNAs seem to suppress cancerogenesis through p21 accumulation and cell cycle arrest. In another recent study, Hino et al. [30] published that human miR-194 expression is induced by the transcription factor HNF-1α promoting intestinal epithelial differentiation. They identified two genomic clusters in the human genome either encoding miR-194-1 and miR-215 on chromosome 1 or encoding miR-194-2 and miR-192 on chromosome 11 [30]. In contrast to human, both porcine clusters are 100% identical encoding only miR-194 and miR-215. In our microarray experiments, miR-194 and miR-215 showed the same expression pattern along the entire intestine, underlining the fact that both may derive from one transcript. However, the expression was highest in duodenum and proximal jejunum compared with other loci. These results are in good concordance with the data derived from human studies. On the one hand, reorganization of the intestine at weaning is associated with high proliferative activity contributing to an increase in crypt depth and villus height [11]. Consistently, in distal parts of the intestine we determined diminished expression of miR-194 and miR-215, respectively pointing to a higher proliferative activity in the distal parts. On the other hand, in the proximal parts of the small intestine particularly a high level of differentiation rather than proliferation takes place at weaning in order to adapt to the new nutritional conditions by acquiring new transport functions. The pathway analysis revealed mainly cell signaling networks to be affected that control differentiation e.g. the mTOR pathway. In addition to regulating cell growth and proliferation, this pathway was reported to promote epithelial morphogenesis and differentiation in vertebrates [31]. Consequently, increased expression of miR-194 and miR-215 in duodenum and proximal jejunum are probably involved in promoting epithelial differentiation in order to replace cells that differ e.g. in transport functions.

Our microarray experiments moreover revealed miRNA clusters expressed ubiquitously in all intestinal loci and others, which are locus specific. The gene cluster composed of MDM288 and miR-214 belongs to the latter and was identified to be upregulated in both colonic loci. The pathway analysis of this particular cluster indicated an impact of these two miRNAs on cell cycle regulation. Accordingly, miR-214 expression was recently shown to be directly linked to cell cycle rate. Decembrini and colleagues showed that miR-214 is one of four miRNAs that are highly expressed in fast cycling retinal progenitors in *Xenopus *governing cell fate decision and developmental timing [32]. In addition to miR-214 and MDM288, miR-19b, miR-23a, miR-24, miR-30b possessed increased expression in colon compared with other parts of the porcine intestine. Interestingly, in another epithelial cell model it was shown that miR-19, miR-23 and miR-24 contribute to lower cell growth when inhibited [33]. These miRNAs seem to be part of a colonic cellular network promoting physiological cell turnover in colon during weaning as discussed above. The porcine intestinal lymphoid tissue comprises two types of Peyer's patches: multiple discrete patches in the jejunum and a long and continuous patch in the ileum [22]. Interestingly, porcine miRNAs highly expressed in these loci (cluster V) may target immune cell differentiation shown by pathway analysis, e.g via targeting the Notch and Wnt signaling. It was recently shown that activation of Notch signaling promotes differentiation of conventional dendritic cells from hematopoietic progenitor cells via activation of the Wnt pathway [34]. The mentioned miRNAs seem also to influence mucosal innate immunity by affecting the anti-proliferative functions of PE-1/METS in macrophage differentiation. The concerted action of emphasized miRNAs and other identified local clusters described here represents the first milestone to understand the complex gene regulation in the developing intestine of a mammal with a close phylogenetical relation to humans.

## Conclusions

Here, we report the first comprehensive miRNA expression analysis along the entire intestine of a mammal. A considerable number of the reported miRNAs expressed in the porcine intestine show low conservation among mammals. These miRNAs may be regarded as porcine specific. On the other hand, the reported miRNAs may share orthologs in other mammals such as human still to be discovered. Our microarray experiments along the entire intestine revealed different clusters of miRNAs, which are expressed in a locus specific manner. This underlines their specific regulative impact on locus specific cellular processes. Future research will highlight distinct functions of the identified miRNAs in biomedical research deciphering cellular reorganization during juvenile intestinal development.

## Methods

### RNA isolation from porcine intestinal tissue

Intestinal samples (~ 2 cm circle segments) were taken from duodenum, proximal and distal jejunum, ileum, ascending and transverse colon of four 31 days old healthy piglets (EUROC × Pietrain) as described earlier [26]. The study was performed following internationally recognized guidelines and was approved by the local animal welfare committee of the Federal Ministry of Consumer Protection, Food and Agriculture (No. G0037/02). The piglets were weaned at the age of 28 days. Samples were quick-frozen in liquid nitrogen and stored at -80°C. In order to obtain representative measurements in each intestinal locus, three cross sections of approximately 2 mm out of the 2 cm segment of frozen intestine were examined. These 3 sections were pooled and total RNA was isolated from samples using an automated homogenizer (FastPrep Instrument, MP Biomedicals, Heidelberg, Germany) and the mirVana miRNA Isolation Kit (Applied Biosystems, Darmstadt, Germany), according to the manufacturer's protocol. The RNA quality and quantity of all samples were proven using the Agilent 2100 Bioanalyzer and the RNA 6000 Nano Kits (Agilent, Waldbronn, Germany) and the Nanodrop 1000 Spectrophotometer (Thermo Scientific, MA, USA).

### Solexa sequencing of pig intestinal small RNAs

Small RNAs from the pig total RNA samples were prepared for Solexa sequencing as follows: ~10 μg total RNA were size-fractionated by Novex 15% TBE-Urea gel (Invitrogen GmbH, Karlsruhe, Germany) and RNA fragments of length between 20 and 30 bases were isolated. The purified small RNAs were then ligated with 5' adapter (Illumina, CA, USA). To remove unligated adapters, the ligation products (40-60 bases in length) were gel purified on Novex 15% TBE-Urea gel. Subsequently, the RNA fragments with the adapter at the 5' end were ligated with 3' adapters (Illumina). After gel purification on Novex 10% TBE-Urea gel (Invitrogen), RNA fragments with the adapters at both ends (70-90 bases in length) were reverse transcribed and the resulting cDNA was subjected to 15 PCR cycles. The amplification products were loaded on Novex 6% TBE gel (Invitrogen) and the gel band containing 90- to 100-bp fragments was excised. The purified DNA fragments were used directly for cluster generation and 36 cycles of sequencing analysis using the Illumina Cluster Station and 1G Genome Analyzer following manufacturer's protocols. Sequencing reads were extracted from the image files generated by Illumina 1G Genome Analyzer using the open source Firecrest and Bustard applications (Illumina).

### Computational analysis of deep sequenced small RNAs

The deep sequencing reads were clipped of 3' adapters and mapped to the pig genome as previously described [35], except that no edits were allowed in the mapping. In total, 1,422,397 reads mapped perfectly to the genome. The Sino-Danish pig genome assembly was used for the analysis [36]. Only genome traces that were minimum 200 nucleotides long and with less than 20% uncalled nucleotides were considered. Because it is known that miRNA mature and star sequences do not occur often in metazoan genomes, deep sequencing reads that mapped perfectly to the genome five times or more were disregarded for purposes of miRNA prediction. The remaining reads were used as guidelines for excising potential precursor sequences from the genome, and these were filtered and scored by the miRDeep core algorithm as previously described [18], except that only mammalian mature miRNAs from miRBase version 13 were used for purposes of seed conservation. miRDeep miRNA candidates that had a score of -3.5 and higher were used in the downstream analysis. Further, since the current pig genome assembly has imperfect coverage, we employed a conservation-based approach to produce a set of low-confidence novel pig miRNAs. First, we identified all sequences that were perfectly represented by ten or more reads in the data. Then we identified mature miRNAs from miRBase Release 14 that matched one or more of these sequences, allowing for up to two edits. These miRBase miRNAs were flagged as possible orthologs and each was assigned a pig miRNA sequence identical to the most abundant matching read sequence. No primate specific miRNA orthologs were flagged, suggesting the absence of contaminations.

### Microfluidic miRNA microarrays

Microfluidic microarray experiments were performed using customized Geniom biochips (febit). Customized microarrays were synthesized with the Geniom One device (febit) applying febit's standard shortmer kit for oligonucleotide synthesis.

The light-activated in situ oligonucleotide synthesis using a digital micromirror device was performed within the Geniom One instrument on an activated three-dimensional reaction carrier consisting of a glass-silicon-glass sandwich (biochip). Using standard DNA synthesis reagents and 3'-phosphoramidites carrying 5'-photolabile protective group, oligonucleotides were synthesized in parallel in eight individually accessible microchannels (referred to as microarrays) of one biochip. Prior to synthesis, the glass surface was activated by coating with spacer to facilitate probe-target interaction and to avoid probe-probe interface [37]. Two different microarray designs were used. The first one included only the 399 miRDeep predicted mature miRNAs and their star sequences in seven replicates allowing highly accurate validation of the miRDeep prediction plus 16 spike-in controls. The second design consisted of 1117 features in triplicate, including the identified conserved porcine mature miRNA sequences (18 mapping to the porcine genome and another 124, which did not map), 74 known porcine and 885 human miRNAs from miRBase (13.0) and 16 spike-in controls.

For microarray experiments, intestinal total RNA samples from different loci of four subjects were isolated independently and respective pools of total RNA samples were prepared. All pools underwent a second quality control to determine the quality and quantity using the Agilent 2100 Bioanalyzer and the RNA 6000 Nano Kit according to the manufacturer's instructions. For each microarray 250 ng of total RNA were suspended in 25 μl of febit's proprietary miRNA hybridization buffer. The hybridization was performed automatically for 16 h at 42°C using the Geniom RT-Analyzer (febit). After stringent washing a microfluidic-based extension assay was performed to label the miRNAs using biotinylated nucleotides [38]. After washing, biotinylated nucleotides were detected by streptavidin-phycoerythrin and signal recognition and calculation were done automatically within milliseconds. The background of the chips was effectively corrected by global background subtraction and inter-array effects were corrected by variance stabilizing normalization [39].

Clustering and heatmap analysis of normalized data was performed using the TM4 software [40] and Venn diagrams were created applying the program VennMaster (Version 0.37.3) available at http://www.informatik.uni-ulm.de/ni/staff/HKestler/vennm/doc.html. Visualization of the RNA secondary structure was performed using the RNAfold web server [41]. Pathway analysis of identified miRNAs was performed by firstly predicting potential targets using Targetscan Release 5.1 [20]. For target analysis of non conserved porcine miRNAs the tool Targetscan Custom was applied using the seed sequence of the identified miRNA. Predicted targets were used for pathway analysis by means of the web based tool DAVID [21] and considering affected BioCarta and KEGG pathways.

## Authors' contributions

SS conceived of the study, performed experiments and analyses, wrote and edited the manuscript. MF accomplished the miRDeep analysis. JS and LH collected the porcine intestinal samples, carried out experiments and contributed to data analysis. WC performed the deep sequencing. AK and PFS designed and performed the microfluidic microarray experiments. NR and RE contributed to the writing of the manuscript and experimental design. All authors read and approved the final manuscript.

## Supplementary Material

Additional file 1 **Identified porcine miRNAs by miRDeep**. Hairpin forming precursors and mature porcine miRNAs were identified from deep sequencing data using the miRDeep algorithm. The table consists of 399 miRDeep scored molecules including assigned porcine miRNAs. Click here for file

Additional file 2 **Identified conserved porcine mature miRNAs by homology search**. Mature porcine miRNAs were identified from deep sequencing data by performing a search for conserved miRNAs among the sequencing reads. Click here for file

Additional file 3 **List of validated novel porcine miRNAs by means of customized microarrays or homology search**. 201 apparently novel porcine miRNAs out of 399 miRDeep predicted sequences were identified either by means of microarray or by homology search. Click here for file

Additional file 4 **miRNAome of the entire porcine intestine identified by means of customized Microarrays**. Background subtracted and variance stabilization normalized (VSN) signal intensities of all samples (duodenum, proximal and distal jejunum, ileum, ascending and transverse colon as well as the common reference) is shown as tab delimited text file. Click here for file
